# The effect of Cu nanoparticle adding on to epoxy-based adhesive and adhesion properties

**DOI:** 10.1038/s41598-020-68162-4

**Published:** 2020-07-06

**Authors:** Necati Ataberk

**Affiliations:** Mechanical Engineering Department, Necmettin Erbakan University, Konya, Turkey

**Keywords:** Mechanical engineering, Nanoscience and technology

## Abstract

Epoxy-based adhesives are widely used for repairing or jointing the metal sheets in the industry. Because of their superior mechanical properties, the metallic nanoparticles can be selected as the additive of the epoxy adhesive. The strength of the Cu nanoparticles (CuNPs) can be expected to improve the mechanical properties of neat epoxy. In this study, CuNPs were added at various weight ratios, such as 1, 2, 5, 10, 15, and 20% into the epoxy resin adhesive. Tensile tests of the dog-bone specimens and the lap-shear tensile tests of the single lap joints were performed for obtaining the mechanical properties. In order to investigate the failure mechanisms, the fractured surfaces of the tensile test samples and adhesively joined sheets were imaged by using a Scanning Electron Microscope. The thermal properties of the adhesives were obtained by using Thermo Gravimetric Analysis and Differential Thermal Analysis. The mechanical and thermal properties of epoxy resin adhesive were improved by adding the CuNPs. The best adding ratios of CuNPs into epoxy were obtained by both mechanical and thermally point of views. As a result of this study, 15 wt% the ratio of Cu nanoparticle adding into the epoxy-based adhesive is suitable for improving the mechanical properties. On the other hand, 20% is the proper Cu nanoparticle adding ratio for the thermal properties improving.

## Introduction

Bonding of the parts made by similar and/or dissimilar materials is widely used as an industrial processes^1, 2^. Bonding by an adhesive is widely used in many areas of the industry, from the aerospace to medical applications^3–6^. Because of their superior physical, chemical, electrical and mechanical properties, these types of joints are preferred by many appliers from the engineers to physicians^7, 8^. Many adhesive materials are using for these types of bondings, such as polymers, metals, etc. One of them is the epoxy resin adhesive. Many researches have been published about the epoxy and micro or nano additives of it^9–12^. One of the application areas of the epoxy-based adhesive is to repair or patch the parts^13^. For example, the outer surface of an airplane body can be damaged by the impact of an object while it flies at high speed. To repair these types of parts, a proper adhesive that is compatible with the outer surface material of the airplane is needed^14^. These types of applications can also be met in many industrial practices. Adhesive joints are expected to behave like having similar mechanical properties of the main material. For this reason, the mechanical properties of epoxy adhesives must be improved by adding some additives when applied on to metallic surfaces^15^. The mixture of the epoxy resin and its curing agent exhibits as a brittle behavior after the mixture cured. But some applications of these types of adhesives are expected to have ductile behavior, especially the metal–metal joints^16, 17^. The mechanical properties of the epoxy resin adhesive can be improved by some additive materials^18–20^. Both the size and type of additives can be enhanced the superior properties to matrix^21–23^. Macro, micro, or nano-sized particles have different properties and effects from their bulk form^24^. For example, nano-sized metallic particles have better mechanical properties^25, 26^. The mechanical or physical property of the epoxy resin adhesive can be improved by adding several metallic nanoparticles. The higher weight ratio of the additives can be affected negatively to composites because of the difficulty of these additives dispersed the homogeneously into the matrix. Also, the higher additive ratios can be responsible for the lower adhesion between the matrix and the additive.

Adhesive joints can be used at various temperatures. Durability and strength at extreme temperatures have always been a significant limitation of adhesives^17^. When repairing some electronic parts by using epoxy-based adhesives, they can be conserved from the adverse effects of higher temperatures of soldering or welding^27^. Adhesive materials can consist of several number of components. Similar to composite materials, these materials can be mentioned in two parts, the matrix, and the additives. Selecting of the matrix and additives is related to the adherent material surfaces. Epoxy-based adhesives can be used on many adherent materials such as polymers, wood, and metals, etc. Some particles can be used as the additive of the epoxy-based adhesives^28–30^. CuNPs are used in many areas as alloyed or alone instead of their bulk or micro-sized form. Because of the ductile behavior of the Cu, the ductility of the epoxy can be enhanced by adding the CuNPs.

This study is about the application of CuNPs into the epoxy-based adhesive for industrial bonding applications. Al (aluminum alloy) 2024-T3 sheets are jointed by using the epoxy-based, and CuNPs added adhesive. CuNPs content was selected as 1, 2, 5, 10, 15 and 20% in weight ratios. Al sheets used in this study are widely used in industry, especially in the aircraft industry. By adding the CuNPs, the adhesive is expected to improve its mechanical properties. However, the mechanical properties can be affected negatively for higher weight ratios by adding CuNPs. This negative effect can also be about micro or higher sized powders^31^. For this reason, the nano-sized particles were selected as additive of adhesives. The expected usage area of this adhesive is the repair or manufacturing of a machine or airplane parts. Lap-Shear and tensile tests, Thermo Gravimetric Analysis (TGA), Differential Thermal Analysis (DTA), and Scanning Electron Microscopy (SEM) imaging were performed for obtaining the adhesives and joint’s properties.

## Experimental

### Materials

**Figure 1:**
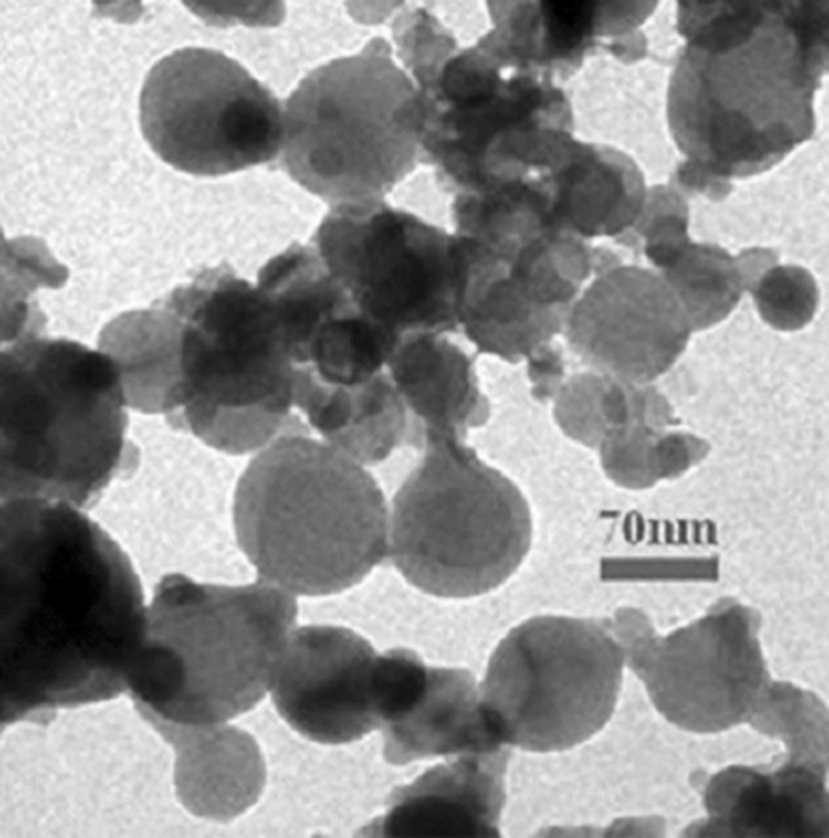
TEM image of Cu nanoparticles.

Epoxy resin (MGS® L285 Bisphenol A (BPA) Epoxide value/100 g is 0.59–0.65) and its curing agent (MGS® H285) is the type of cyclo-aliphatic amine at 70–90% and polyoxyl alkyl amine mixture at 10–30% produced by Hexion (in Spain) and purchased from Dost Kimya (in Turkey) was used in this study. The densities of the epoxy resin/hardener are about 1,180–1,230/940–970 kg/m^3^, and the viscosities are 600–900/50–100 mPa s at 25 °C and the gelation time of the epoxy-hardener system is approximately 1 h. The epoxy resin and curing agent were mixed 100/40 in weight ratio as recommended by the manufacturer. This ratio can express in terms of the stoichiometric ratios as 71.4–28.6%. Al alloy adherent sheets purchased from TAI (Turkish Aircraft Industry in Turkey) were used as adherent material in a 1.6 × 25 × 101.6 (in mm) dimensions. CuNPs used in this study were purchased from Genbiotek Biosystem Laboratory Company in Turkey. The shape and size of the CuNPs are shown in Fig. 1. CuNPs have various diameters, as shown in Fig. 1. The mean diameter of CuNPs is 70 nm. It is expected that this particle size variation positively affects the properties of the adhesive.

### Methods

In this study, seven types of epoxy-based adhesives were prepared, and the contents of the manufactured adhesives are presented in Table 1. To achieve a good joint between the adhesive and adherent’s surfaces, chemically etching process in accordance with ASTM D2651 and phosphoric anodization with ASTM D3933 were performed. Epoxy resin and CuNPs were stirred by using Bandelin HD 2,200 ultrasonic probe homogenizer (manufactured in Germany) in an ice bath for 30 min at 20 kHz, 70 W. %25 power level was set on the stirring device. Stirring was discontinued every 10 min in order to avoid overheating and to minimize the damage on nanoparticles because of the ultrasonic vibrations. The mixing procedure was repeated for getting the homogeneous mixture^32^. As recommended by the manufacturer, the mixture temperature was limited between the + 10 and + 50 °C in an ice bath. Then the mixture was stayed at vacuum for 10 min at 0.02 MPa to avoid the air bulbs. At last, the hardener was added in the mixture at 100/40 weight ratio as advised by the manufacturer and mixed mechanically for 10 min. Mixtures were stayed for 5 min in a vacuum at 0.05 MPa for removing the bubbles. Produced adhesive material was used for preparing both the dog-bone tensile test specimens and the Single Lap Joint (SLJ) tensile test specimens.

**Table 1:** Abbreviation and contents of the adhesive samples.

Sample	CuNPs ratios (%) in weight
NE (neat epoxy)	0
1CuE	1
2CuE	2
5CuE	5
10CuE	10
15CuE	15
20CuE	20

Tensile test samples were poured in an open mold. The open steel mold was prepared and dimensioned according to ASTM D638-10, and the mold cavity was calculated. Lap joint tests were performed according to ASTM D1002-10. The cavity between the two adherents (0.2 × 12.7 × 25.4 in mm) was calculated. The adhesive amount was adjusted that all cavities could be filled.

Various methods can be applied to producing the bulk form of specimensf^33^. The mechanical properties of the adhesive joints were obtained in accordance with the ASTM F2255 standard, and the dog-bone tensile test specimens were prepared in accordance with the ASTM D638-10 for obtaining the mechanical properties of adhesive materials. For reliability of the results, five specimens that have the same contents were tested. Tensile tests were performed on these specimens for obtaining the mechanical properties of the adhesives by using the Shimadzu AGS X series (made in Japan) tensile test machine at room conditions (25 °C and 60% relative humidity). Both the axial and the transversal displacements were measured by using Epsilon 3,560 model bi-axial extensometer during the tensile tests.

**Figure 2:**
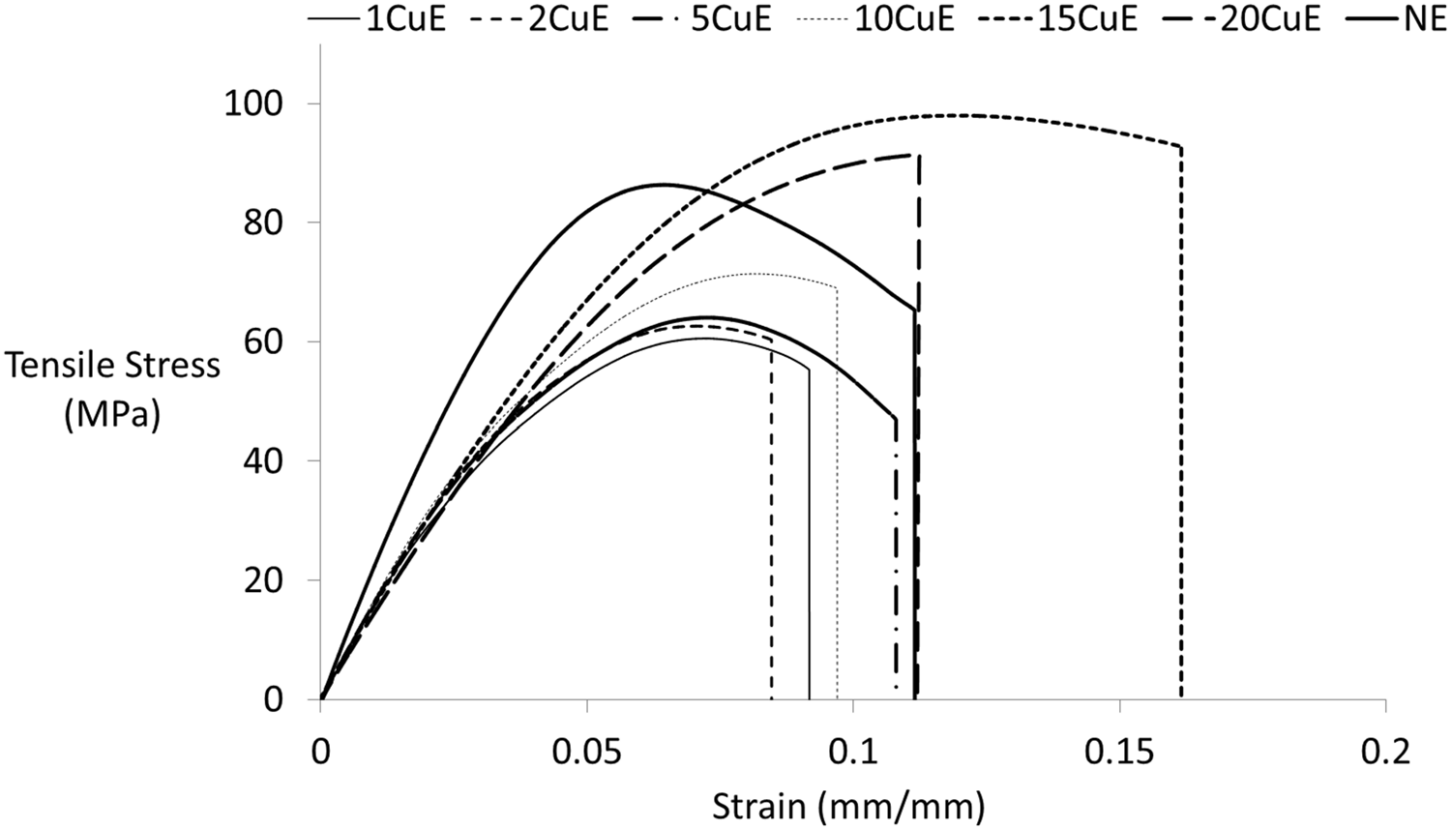
Tensile test results at a constant crosshead speed of 1 mm/min.

The tensile test results are presented in Fig. 2. The area under the stress–strain curve determines the material toughness^34^ that the total energy of the materials carried out. These values were calculated and presented in Table 2.

**Table 2:** Mechanical properties of adhesive materials.

Specimen	Modulus of elasticity (GPa)	Toughness × 10^–3^ J/mm^3^	Poisson’s ratio	Tensile strength (MPa)	Maximum strain % (mm/mm)
1CuE	2.895 ± 0.21	0.253	0.259	64.19 ± 3.59	5.40 ± 0.21
2CuE	3.050 ± 0.58	0.244	0.269	62.69 ± 1.27	6.15 ± 1.29
5CuE	3.431 ± 0.37	0.360	0.246	64.63 ± 1.04	6.05 ± 0.43
10CuE	3.327 ± 0.10	0.329	0.228	71.00 ± 1.02	5.33 ± 0.46
15CuE	3.991 ± 0.16	0.340	0.239	96.34 ± 2.05	8.88 ± 0.63
20CuE	3.747 ± 0.36	0.222	0.212	83.66 ± 7.62	10.56 ± 3.09
NE	3.846 ± 0.11	0.405	0.232	86.42 ± 3.51	10.27 ± 0.75

**Figure 3:**
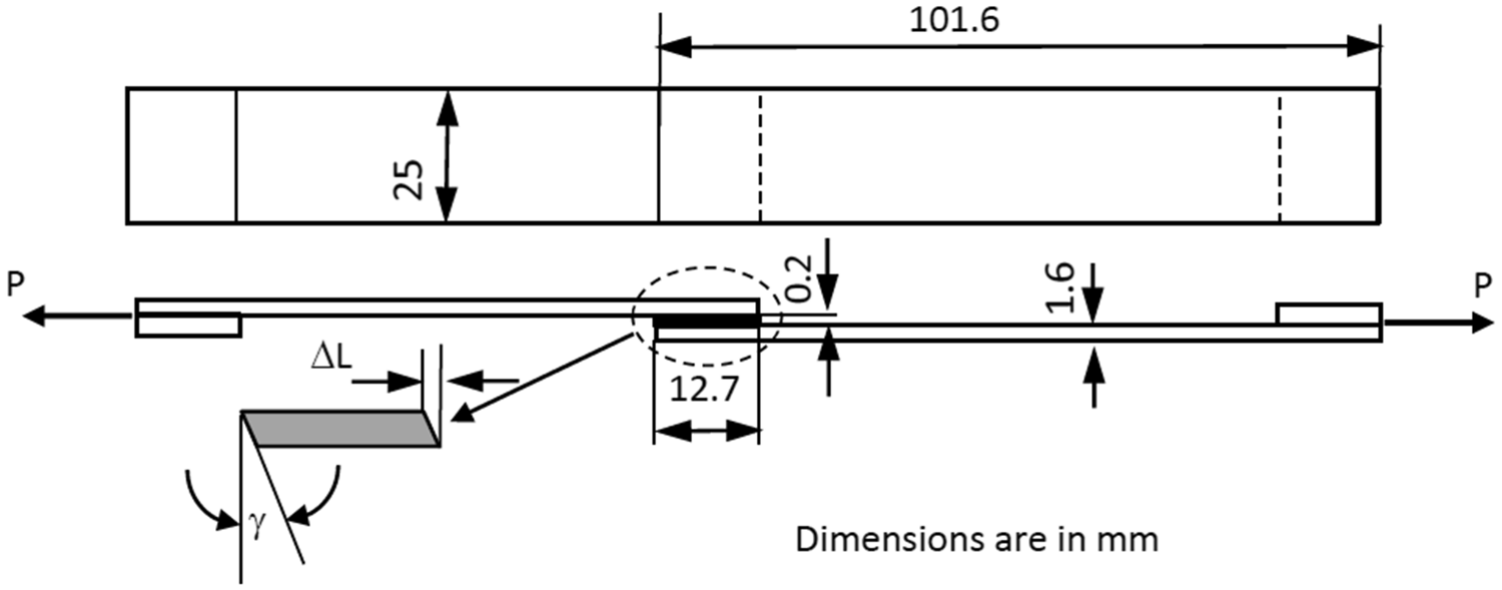
Schematic view of the Lap-shear tensile test specimen.

The accuracy of the results of strength tests of adhesive bonds depends on the conditions under which the bonding process is carried out. For this reason, the Single-Lap-Joint tensile tests in accordance with ASTM D1002-10 were performed for obtaining the adherents’ bonding properties by using Shimadzu AGS X tensile test machine at a constant crosshead speed of 1 mm/min. The metal pads at grip areas of the samples were placed for eliminating the bending effect during the tensile test. Following ASTM D3933-98, chemically prepared adherents having a bondline length of 12.7 mm were bonded together, as shown in Fig. 3.

The thickness of the SLJ (*t* = 0.2 mm) was provided by using the shims of proper thickness at grip areas of the tensile tests as seen in Fig. 3. Using these shims, adhesive thicknesses of 0.2 mm were obtained reproducibly. According to the manufacturer’s instructions, there is a waiting period of maximum 30 min between the preparation of the adhesive and its application to the surface. In this study, the adhesive was poured onto the aluminum surface in approximately 15 min and distributed evenly. The mean values of the shear stresses (*τ*) and shear strains (*γ*) were calculated by using Eqs. (1) and (2), respectively. Elongation of the bonding region (ΔL) was measured by Epsilon 3,560 extensometer.

1 $$\tau = \frac{P}{w.L}$$

2 $$\gamma = \frac{\Delta L}{t}$$

where the values w, L, *Δ*L, t, and P are the width (25 mm), bondline length (12.7 mm), measured elongation at the jointed region (mm), thickness (0.2 mm) of the joint and the tensile load (N) respectively. The multiply w.L is the area of the shear at the adhesive joint.

**Figure 4:**
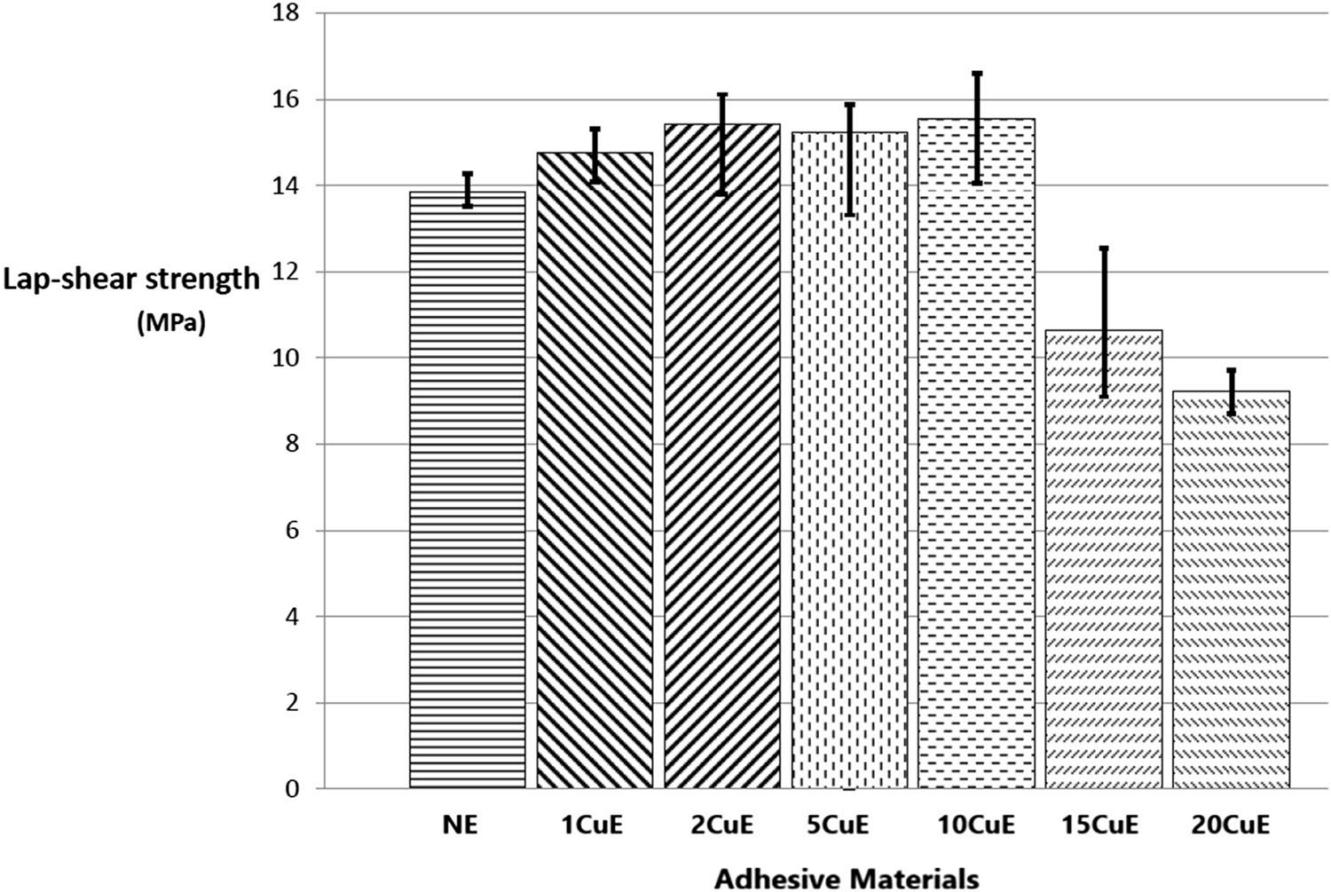
Lap-shear tensile test results.

Shear strength of the joint values calculated by Eq. (1) and given in Fig. 4 and Table 3 are the maximum stress of the SLJ test results.

**Table 3:** Shear strength of single lap adhesive joints.

Specimen	Shear strength (MPa)
NE	13.85885 ± 0.374
1CuE	14.75228 ± 0.618
2CuE	15.41553 ± 1.154
5CuE	15.21939 ± 1.286
10CuE	15.52928 ± 1.270
15CuE	10.64667 ± 1.727
20CuE	9.229956 ± 0.498

**Figure 5:**
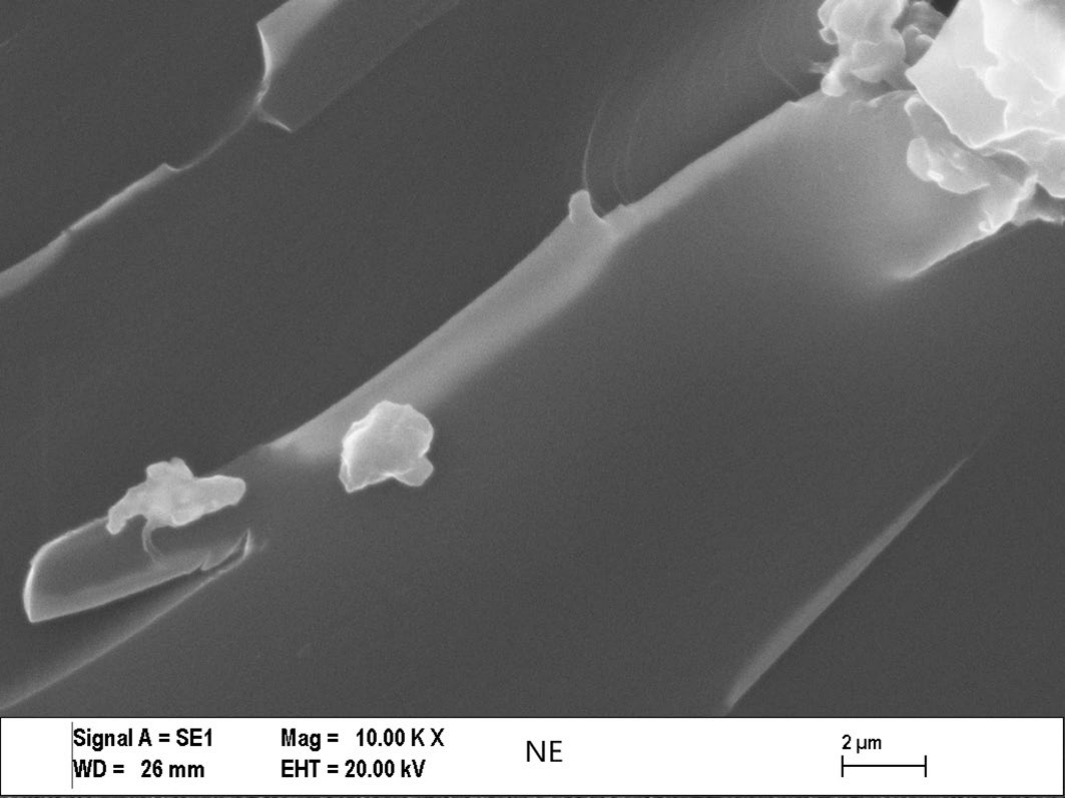
SEM images of the tensile test specimens’ ruptured surfaces of 10KX for NE.

**Figure 6:**
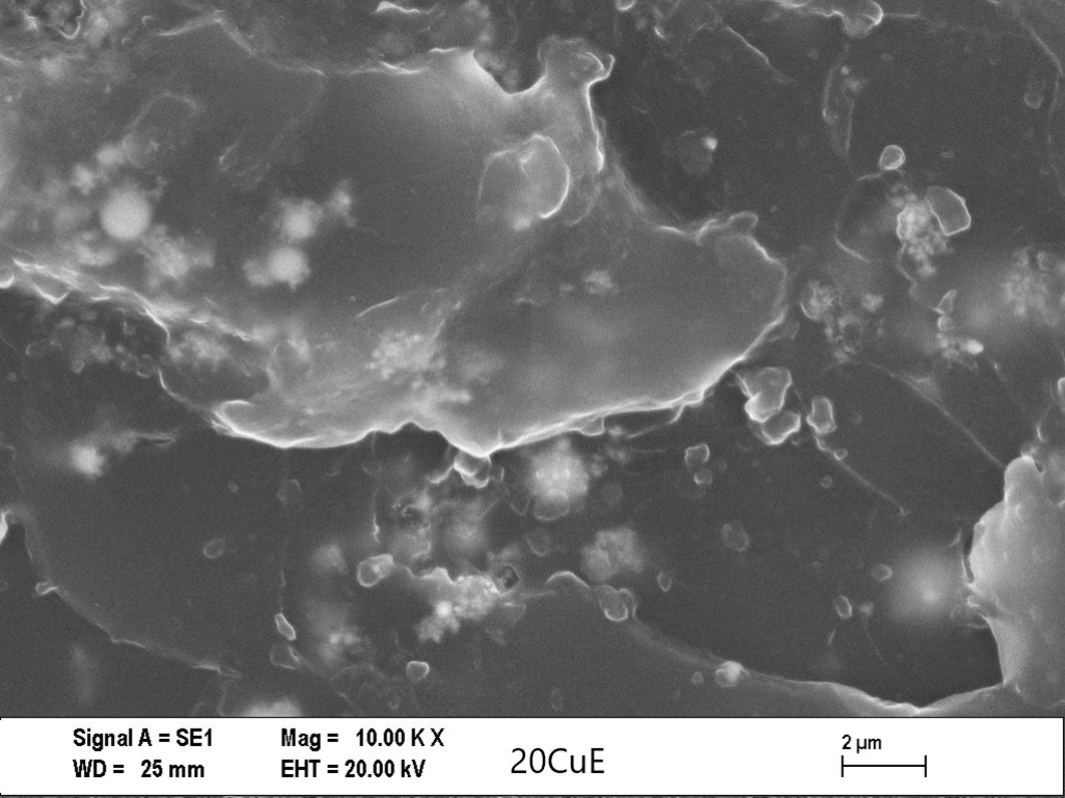
SEM images of the tensile test specimens’ ruptured surfaces of 10KX for 20CuE specimens.

**Figure 7:**
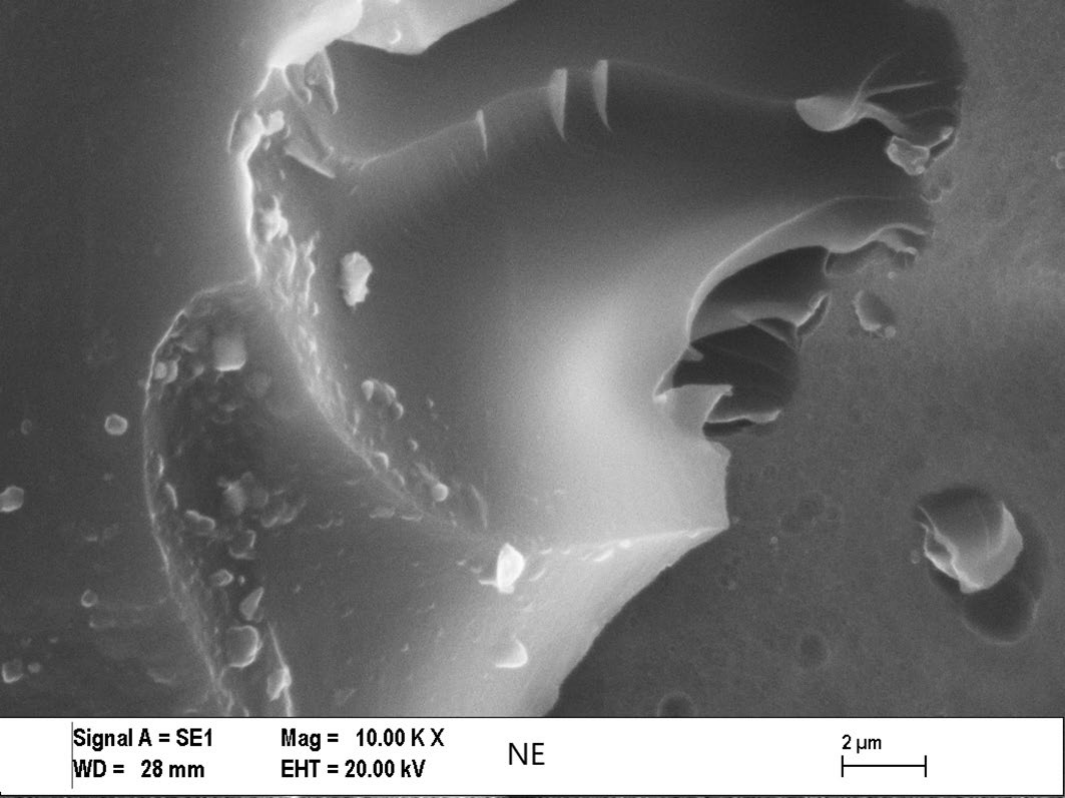
SEM images of the ruptured Single Lap Joint specimens’ surfaces of 10KX for NE.

**Figure 8:**
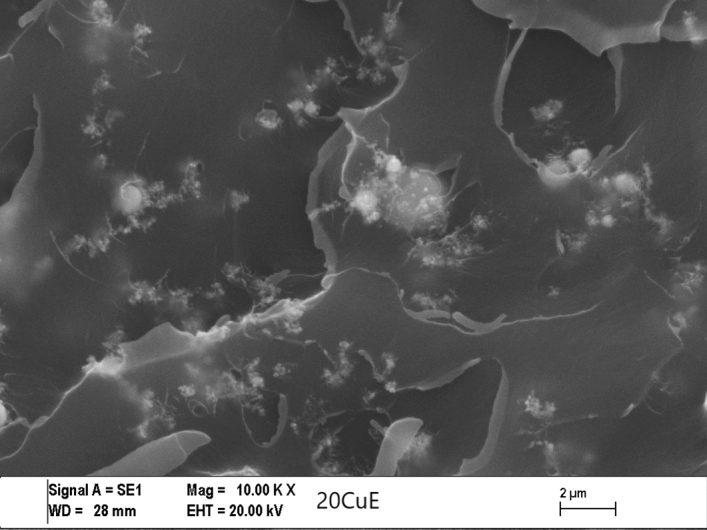
SEM images of the ruptured single lap joint specimens’ surfaces of 10KX for 20CuE specimens.

For investigating the type of failure after tensile tests, both the adhesive’s and adherents’ (Al sheets’) ruptured surfaces were imaged by Zeiss (LEO) 1430VP model Scanning Electron Microscope (SEM) made in Germany. The effect of Cu content on the failure mechanisms for some of the fractured tensile tests and SLJ tensile test specimens and can be seen in Figs. 5, 6, 7, and 8 respectively, at the same magnification. The dispersion of the CuNPs into epoxy also viewed by using SEM after tensile tests. According to SEM images, it can be seen that the nanoparticles were dispersed in the tensile test and SLJ specimens' fractured surfaces. However, some CuNPs agglomerated regions were observed, and the reason for this is thought to be due to the mixing time. Epoxy resin reached higher temperatures after higher mixing times, and it was affected negatively, so the mixing procedure was stopped after every 10 min. Particles and epoxy matrix behaved as an integrated composite material. Almost all ruptures of the samples were the epoxy fracture. This failure may be due to the high strength of CuNPs.

**Figure 9:**
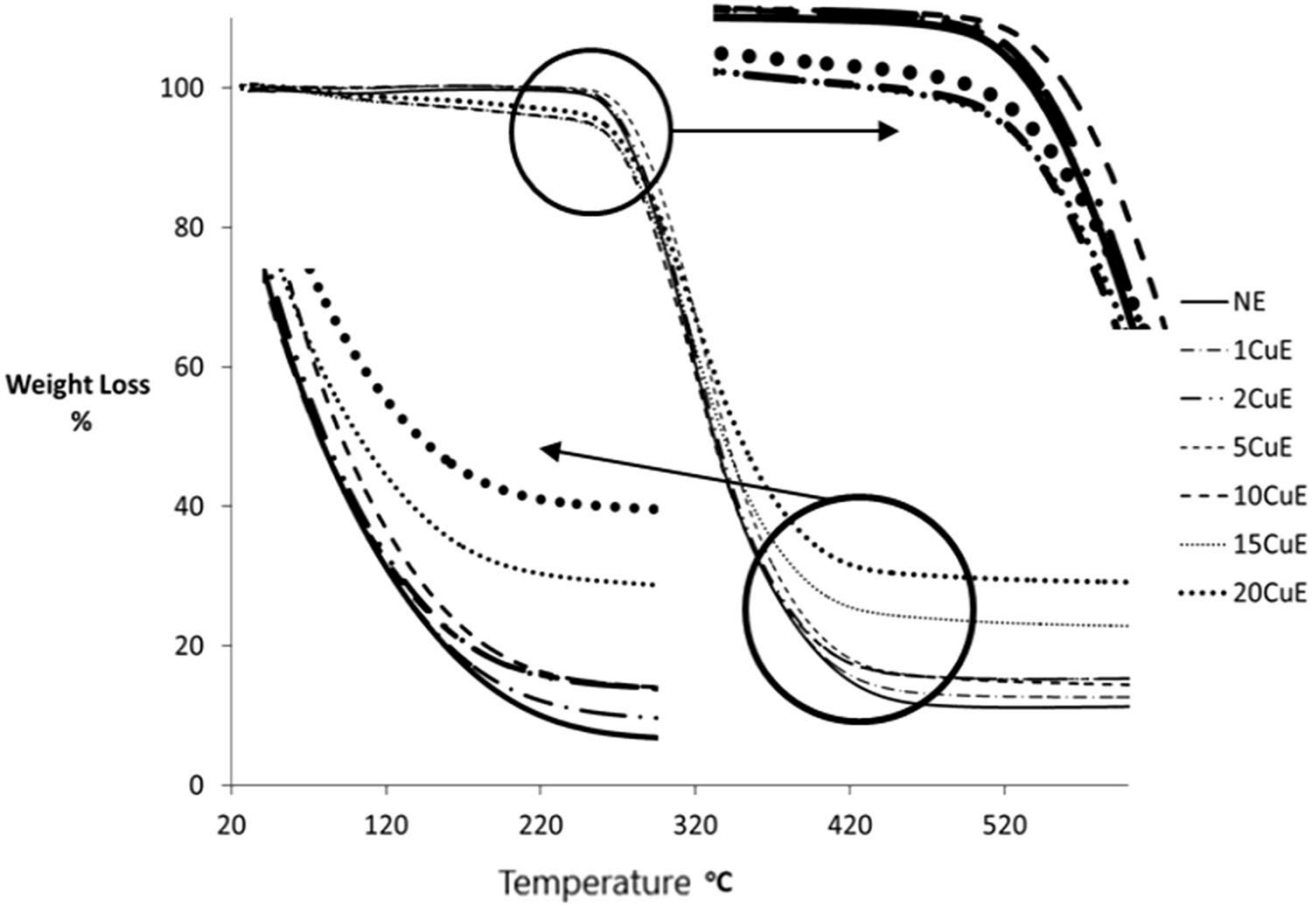
TGA results, % weight versus temperature changes for different adhesive materials.

**Figure 10:**
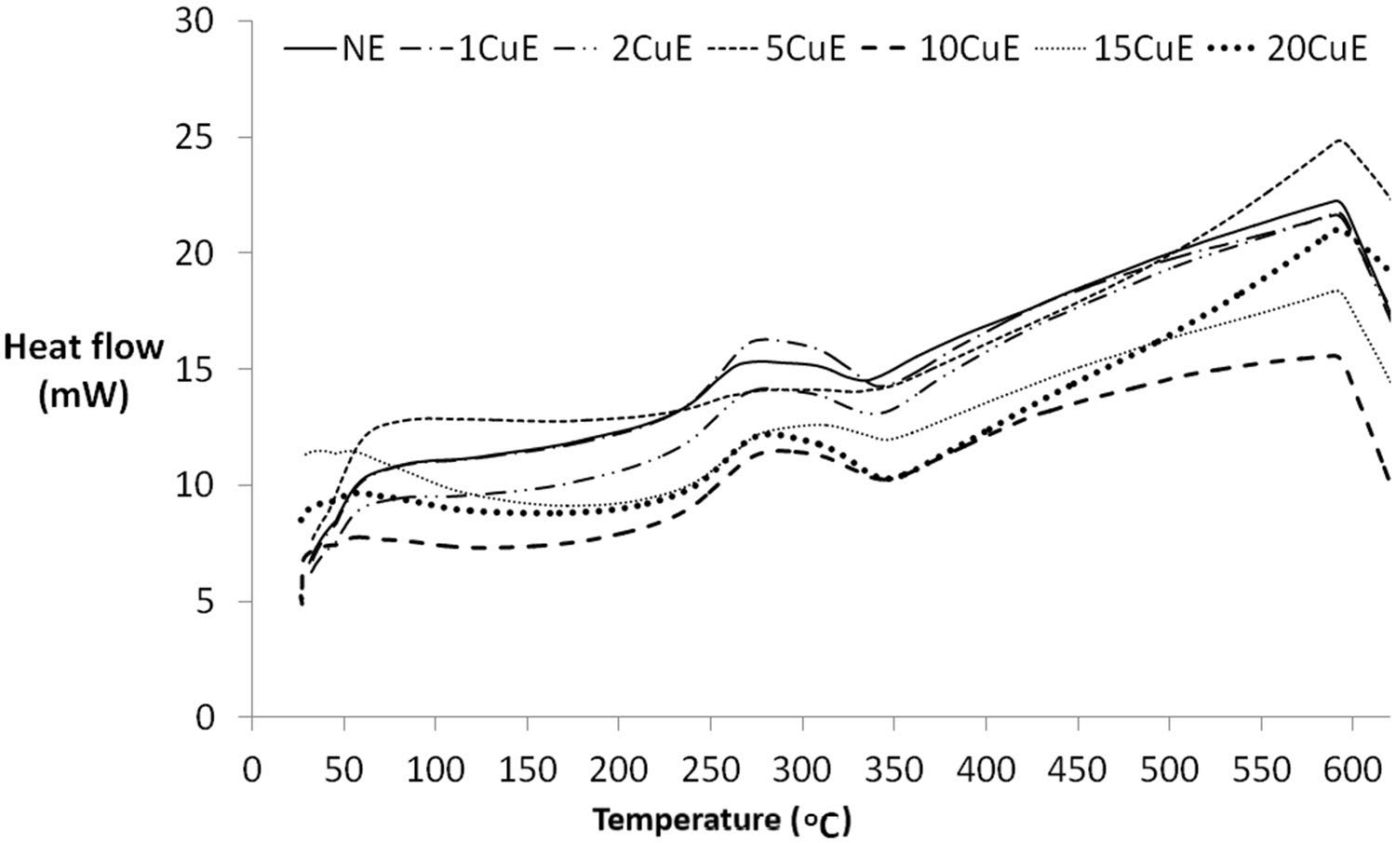
DTA results, heat flow (mW) versus temperature for adhesive materials.

Thermal stability and the decomposition properties of the adhesives were obtained by using the Thermo Gravimetric Analysis (TGA) and the Differential Thermal Analysis (DTA) techniques. TGA results are plotted in Fig. 9. These tests were performed by using Setaram-Labsys DTA and TGA device (made in France). Approximately 8 g of each different one sample was used in thermal analysis. The temperature range was selected 25–650 °C; all tests were performed in a Nitrogen atmosphere, at 10 °C/min temperature rate. By using thermal analysis values, mass lost temperatures are presented in Table 4 for the selected mass lost points, i.e., 5, 20, 70%, and not mass lost conditions. DTA results of the specimens are presented in Fig. 10. In DTA graphs, the picks indicate the endothermic reactions.

**Table 4:** Mass lost temperatures.

Specimen	Mass lost temperatures (°C)				
	Initial	5%	20%	70%	Decomposition temp. (°C)
NE	250.9	270.0	296.3	365.61	590.7
1CuE	250.4	270.9	296.4	365.73	590.5
2CuE	251.5	272.4	298.3	367.65	591.3
5CuE	252.0	277.3	301.8	379.83	592.5
10CuE	250.3	250.9	292.6	365.99	589.5
15CuE	250.0	248.1	294.9	388.56	590.8
20CuE	250.7	260.1	299.7	478.24	592.2

Almost all of the specimens exhibit the endothermic reaction between the temperature range at 225–345 °C. The area under the curve of this temperature range (in terms of heat flow versus time) is named the resultant melting enthalpy value of the specimen. Melting Temperature (T_M_), resultant melting Enthalpy (ΔH), and Glass Transition Temperatures (T_G_) of the samples are presented in Table 5.

**Table 5:** Thermal properties of the specimens.

Specimen	T_M_ (°C)	ΔH (J)	T_G_ (°C)
NE	276.8	12.752	67.1
1CuE	280.4	22.118	66.28
2CuE	280.4	14.826	63.25
5CuE	306.5	4.803	60.85
10CuE	288.4	19.042	58.10
15CuE	308.7	13.651	56.83
20CuE	282.5	20.805	56.23

## Test results

### Mechanical tests

Epoxy-based adhesives exhibit the brittle fracture behavior because of having the cross-links at polymer chains. In this study, almost all of the failures have occurred in adhesive mode and brittle as expected from the epoxy’s brittle nature^35–37^. CuNPs were added at various weight ratios such as 1, 2, 5, 10, 15, and 20%. The mechanical property of the epoxy was changed by adding the CuNPs. A similar study has been demonstrated that there is a relationship between the shear strength and the filler ratios^38^. As shown in Table 2, the highest tensile strength 96.34 ± 2.05 MPa was obtained for 15CuE tensile test specimen while 86.42 ± 3.51 MPa for NE specimen. The Modulus of Elasticity value can determine the rigidity of the material. Modulus of Elasticity was obtained as 3.846 ± 0.11 GPa for the NE, the highest value of 3.991 ± 0.16 GPa was obtained for 15CuE specimen. There is not any considerable rigidity changing between the specimens that contained the CuNPs. The toughness of a material can be considered as the energy that the material can absorb with the effect of external loads until the failure. The toughness values were obtained by calculating the area under the stress–strain curve and not only related to the stress but also the maximum strain values. The toughness of the NE sample was obtained as 0.405 × 10^–3^ J/mm^3^, while the maximum value for the 5CuE sample was 0.360 × 10^–3^ J/mm^3^. This can be explained by the fact that CuNPs are more rigid than epoxies. Another mechanical property of the material is Poisson’s ratio. This value can be defined as the material’s deformation capability in the transverse direction of the loading. The Poisson's ratios were not considerably affected by the Cu nanoparticle adding. The deformation behaviors, both the along and normal to the loading directions, were affected similarly by adding CuNPs. Because of this, the Poisson’s ratios were not varied considerably.

Adherent sheets were bonded by using the produced adhesives, as shown in Fig. 3 to obtain the bonding properties of the adhesives. Because of the sample geometry and the loading direction, Mode II fracture occurred. Deformations of the samples were measured only in the joint region. The mean values of the shear strengths of joints are presented in Table 3, according to SLJ test results. Because of the lower strength (96.34 MPa) [under the yield strength of the adherent sheets (320 MPa)] of adhesive, there was not any plastic deformation observed at adherent sheets after the tensile tests. While the shear strength of the joint by using NE adhesive was obtained as 13.85 ± 0.374 MPa, the highest shear strength of the joint (15.52 ± 1.27 MPa) of the bonding was obtained for 10CuE sample, and the minimum value (9.22 ± 0.498 MPa) was obtained for the 20CuE sample (see the Table 3). It can be mentioned that the CuNPs were improved the bonding property of the 10CuE sample, but for higher adding ratios, the shear strength of the SLJ was affected negatively.

While the maximum tensile strength was obtained for 15 wt% CuNPs added sample, and the maximum joint strength was obtained for 10 wt% CuNP added adhesive. The reason for this can be commented that the joint conditions such as the surface roughness or adherent material were affected by the obtained joint strength value.

### Morphology analyses of the fractured surfaces

SEM analyses were performed for obtaining the morphology of the fractured surfaces. The surface roughness of the CuNPs contented fractured tensile test and lap-shear test samples can be viewed in Figs. 6 and 8, respectively. CuNPs content can be seen in these figures. It can be seen also in Figs. 5, 6, 7, and 8 that the surface roughness of the fractured samples increased by increasing the amount of CuNPs. These results are also compatible with mechanical tests. The fractured surfaces of the tensile test specimens were presented in Figs. 5 and 6. The fractured surface of the NE specimen was different from Cu nanoparticle added samples. Flat surfaces seemed for NE fractured sample. This can be commented on as the brittle fracture of the epoxy. Crack propagation seemed as straight lines for the NE specimen as shown in Figs. 5 and 7, but CuNPs contented samples have different fractured surfaces as shown in Figs. 6 and 8. Morphology of the CuNPs contented samples’ surfaces can be named the rough. This can be commented as the brittle fracture mechanism of the epoxy was changed ductile by adding the CuNPs into epoxy.

### Thermal analysis results

Thermal properties of the adhesive materials were examined by TGA and DTA techniques. Glass transition temperature (T_G_) is one of the thermal properties of a polymeric material. This value limits the using temperature of an adhesive^39^. The maximum T_G_ value (67.1 °C) was obtained for the NE sample while the minimum (56.23 °C) for the 20CuE sample (see Table 5). Similar results (decreasing the T_G_ by adding the particles) were obtained at a previous study for adding the Nano-silica particles^38^. Another thermal property is the decomposition temperature of a polymeric material. Decomposition temperature can be named the limit of the thermal stability of a material. The thermal stability of a material can be observed by TGA results. It can be seen in Fig. 9 that, there was not any considerable loss of mass above the 421.6 °C for the 20CuE sample by using TGA results. At this temperature, the 20CuE sample conserved to 31% of its mass. As expected that the maximum mass loss (88%) occurred for the NE sample at 440.7 °C. The maximum decomposition temperature was obtained for 5CuE as 592.5 °C, while the minimum was 589.5 °C for the 10CuE sample. These results show that CuNPs adding has not significantly affected decomposition temperatures. The melting temperature of the NE sample (276.8 °C) was increased to 308.7 °C for the 15CuE sample by adding the CuNP. Although the highest value of the melting point (308.7 °C) was for 15CuE, it was 282.5 °C for the 20CuE sample.

Temperatures that start and finish the decomposition of a material determine the using temperatures region of the material. This property can be changed by additives in a polymeric material. As seen in Table 4 that there is not any significant change in both the start or finish temperatures of decomposition. At the end of the tests, the residual mass was increased by increasing the CuNPs contents as expected. These residues consist of almost only the CuNP. There is not any considerable loss of mass that occurred until 250 °C for almost all samples while the TGA. After this temperature, the thermal stability was lost speedily till the 400 °C. The maximum of the mass loss start temperature was obtained for the 5CuE sample while the minimum was obtained for 15CuE. After the loss of mass starting temperature, there was a steady, and a swift loss of mass occurred for all samples until the 370 °C.

Loss of mass was continued at fewer for the higher temperatures greater than 370 °C. Above this temperature, the NE sample was lost almost the whole of its mass. The reason for the minimum loss of mass for the 20CuE sample may be the maximum CuNPs content for this sample, and Cu particles which were not showing any change above the decomposition temperature. Decomposition temperatures of the samples were ranging in a constricted interval between 589.5 and 529.2 °C. Above this temperature, almost all samples’ epoxy contents were fully decomposed. There is no considerable change in decomposition (start or finish) temperatures in almost all samples. The reason for this can be CuNPs that were not as effective as at the beginning of the tests.

Another thermal property of an adhesive is the melting energy, and it was obtained by using DTA results. All samples exhibited the endothermic reaction between 225 and 345 °C interval, as seen in Fig. 10. The maximum melting energy (enthalpy) was obtained for the 1CuE sample. Almost all enthalpy values of Cu contented samples’ were greater than the NE sample. The CuNPs absorbed the heat from the beginning of the DTA test to 225 °C and left this heat during the melting interval.

## Discussion

No studies on epoxy-based copper nanoparticle adhesives discussed in this study have been found. In a study^40^ on the effect of silver nanoparticles decorated with boron nitride nanoplates incorporated into the epoxy adhesive on thermal stability, thermal stability increased. These findings are consistent with the results obtained in this study, and shown in the TGA graphics. Dynamic mechanical analyzes were also performed in the same study and it has been shown that it provides optimum mechanical properties for 20% of the particle additive. In this study, this rate was found to be 10 wt% for CuNPs additive. The electrical and mechanical properties of the epoxy adhesive were enhanced by adding MWCNT and Ag nanoparticles^41^. In that study, the epoxy adhesive that consisted of 0.5 wt% MWCNT and 0.5 wt% Ag presented the higher mechanical properties than the neat epoxy.

## Conclusion

This study was carried out to investigate the effect of copper nanoparticle addition on the properties of epoxy adhesives. Epoxy resin is hardened by adding the curing agent. After curing, these types of adhesives exhibit brittle behavior. But also these type of adhesives are used to repair or joint of the metallic components. For this purpose, a ductile property is required from the epoxy-based adhesive. Because of this, CuNPs were added into the epoxy resin adhesive as various weight ratios such as 1, 2, 5, 10, 15, and 20 wt%.

While preparing the samples of this study, neat epoxy was used as a pure form by an ultrasonic mixer, but the composite samples needed to be mixed with CuNP. After the mixing, the samples were kept into the vacuum environment to avoiding the occurred air bubbles while mixing by the ultrasonic mixer. This process could not thoroughly remove the air bubbles in the composite samples. But the neat epoxy samples had not any air bubble. For this reason, some of the mechanical properties obtained by obtained the tensile tests are not values as high as expected. This negative situation could be avoided by mixing the neat epoxy sample by the ultrasonic mixer. But this application was meaningless for the user of the adhesive.

By inspection of the SEM images given, one can see the CuNP were distributed almost homogeneously. But there are small and few agglomerated regions that are also seen in the SEM image. For the more mixing time, the more homogeneous mixture can be reached. In that case, the mixing process may be harmful to epoxy or nanoparticles because of the temperature rising and the mechanical effects of ultrasonic waves.

Modulus of Elasticity of the Neat epoxy was decreased by adding the CuNP. It can be concluded from this result that the ductility of the epoxy-based adhesive was enhanced.

The strength of the adhesive and strength of the joint is the different characteristics of an adhesive material. Maximum tensile strength (96.34 MPa) of the bulk specimen was obtained for 15 wt% Cu nanoparticle added specimen, but the maximum joint strength (15.52 MPa) was obtained for 10% Cu nanoparticle added specimen. After the SLJ tests, almost all of the failures occurred by adhesively (fracture between the adhesive and adherent). This can be commented as because the perfect integration between the adhesive and adherents can not be reached, or the strength of the composite adhesive is higher than the linking between the adhesive and adherents’ surfaces.

These different results can be related to the surface condition of the adherent materials. It can also be concluded that the nanoparticles affected adhesion between the adhesive and adherents surfaces’. The bulk specimen property is the determiner only for the adhesive material. By the point of view of mechanical properties, the 15 wt% the ratio of Cu nanoparticle can be mentioned about the optimum value for the adhesive, but if we use this material as an adhesive 10% weight ratio must be selected. For the higher particle ratios, the interface between the adhesive and adherent gets smaller. Because of this effect, the 10 wt% Cu nanoparticle ratio can be advised as the proper for Al–Al joints.

The modification mechanism of the mechanical properties can be commented as: CuNP have resisted to deformation of epoxy. The deformation of the neat epoxy tensile test sample is higher than almost the nanoparticle added ones. But adding CuNP have affected negatively on adhesion between the adherent surfaces and adhesive.

Another essential property of an adhesive is the thermal stability and the glass transition temperature. Especially for the metallic adherents, the adhesive must conserve its properties during the application temperature range. For the higher Cu nanoparticle ratios into the epoxy resin adhesive, the thermal stability of the adhesive decreased. The reason for this can be imagined as the absorbed heat by the metallic nanoparticles in epoxy at the beginning of the thermal analysis, and then this CuNP behave as a heat source in the mixture. If heat transfer continues, the epoxy matrix reaches over the decomposition temperature. In order to conserve the thermal stability of epoxy, the maximum Cu nanoparticle content must be selected as 20 wt%.
